# Supplementary material for: Effect of Nutritional Deprivation after Sleeve Gastrectomy on Bone Mass, Periostin, Sclerostin and Semaphorin 4D: A Two-Year Longitudinal Study
Source: Nutrients. 2023 Oct 10;15(20):4310. doi: 10.3390/nu15204310 (PMC10610316; doi:10.3390/nu15204310)
Supplement: Supplementary file 1 [file nutrients-15-04310-s001.zip › nutrients-2638318-supplementary.pdf]

Supplemental materials Table S1. Influence of various anthropometric and biological variables on whole-body aBMD variation at 12 months.

		**% relative variation (Δ 12m-baseline/baseline) of the whole body aBMD**					
**Continuous variables expressed as % relative variation (Δ 12m-baseline/baseline)]**	N	Univariate analysis			Multivariate analysis (n=68)		
		$_c\beta$	SE	p-value	$_a\beta$	SE	p-value
**Age at baseline**	71	-0.012	0.029	0.686			
**Weight**	71	-0.009	0.043	0.834			
**BMI**	71	-0.009	0.043	0.825			
**Calcium**	71	0.05	0.115	0.668			
**Phosphorus**	71	0.005	0.019	0.790			
**iPTH**	71	-0.023	0.011	0.038	-0.024	0.011	0.024
**25(OH)D3**	71	0.01	0.009	0.279			
**Albumin**	69	0.057	0.049	0.244			
**Osteocalcin**	71	-0.01	0.006	0.079	-0.009	0.005	0.085
**CTX**	70	-0.003	0.002	0.107			
**Periostin**	71	-0.029	0.016	0.086			
**Sclerostin**	71	-0.004	0.018	0.820			
**Semaphorin**	71	-0.015	0.021	0.469			
**Categorical variables at baseline**							
**Gender** **Male**<br>**Female**	16/71<br>55/71	2.304<br>-	0.787<br>-	0.005	2.63	0.754	0.001
**Hypertension** **Yes**<br>**No**	29/71<br>42/71	-0.122<br>-	0.709<br>-	0.864			
**Type 2 diabetes mellitus** **Yes**<br>**No**	13/71<br>58/71	-0.003	0.901	0.997			
**Sleep apnoea** **No**<br>**Yes**	27/71<br>44/71	-0.946<br>-	0.709<br>-	0.187			

Legend: BMI: body mass index, iPTH: intact parathyroid hormone; 25(OH)D3: 25 vitamin D; CTX: type I-C telopeptide breakdown products. Δ 12m-baseline/baseline represents the % relative difference between values at 12 months and baseline. $_{c}\beta$: crude standardized coefficients; $_{a}\beta$: adjusted standardized coefficients; SE: standard error. P-values were from linear regression analysis.

Supplemental materials Table S2. Influence of various anthropometric and biological variables on whole-body aBMD variation at 24 months.

		% relative variation (Δ 24m-baseline/baseline) of the whole body aBMD					
**Continuous variables expressed as % relative variation (Δ 12m-baseline/baseline)**	**N**	**Univariate analysis**			**Multivariate analysis (n=55)**		
		**$_c\beta$**	SE	**p-value**	**$_a\beta$**	SE	**p-value**
**Age at baseline**	58	-0.096	0.059	0.108	-0.086	0.051	0.096
**Weight**	58	-0.067	0.075	0.376			
**BMI**	56	-0.073	0.075	0.334			
**Calcium**	58	-0.136	0.221	0.542	-0.452	0.215	**0.041**
**Phosphorus**	58	0.037	0.038	0.329			
**iPTH**	58	-0.048	0.013	**0.001**	-0.046	0.0144	**0.003**
**25(OH)D3**	58	0.018	0.012	0.135	0.022	0.011	**0.044**
**Albumin**	56	0.051	0.085	0.551			
**Osteocalcin**	56	-0.043	0.012	**<0.001**	-0.031	0.011	**0.005**
**CTX**	57	-0.005	0.004	0.169			
**Periostin**	57	0.027	0.032	0.402			
**Sclerostin**	57	0.119	0.041	0.005	0.058	0.037	0.121
**Semaphorin**	53	-0.032	0.025	0.218			
**Categorical variables at baseline**							
**Gender** **Male**	14/58	-0.120	1.670	0.943			
**Female**	44/58	-	-	-			
**Hypertension** **Yes**	26/58	-2.013	1.411	0.159			
**No**	32/58	-	-	-			
**Type 2 diabetes mellitus at baseline**							
**Yes**	14/58	-1.652	1.655	0.323	3.275	1.602	0.047
**No**	44/58	-	-	-			
**Sleep apnoea** **No**	22/58	1.114	1.465	0.450			
**Yes**	36/58	-	-	-			

Legend: BMI: body mass index, iPTH: intact parathyroid hormone; 25(OH)D3: 25 vitamin D; CTX: type I-C telopeptide breakdown products. Δ 12m-baseline/baseline represents the % relative difference between values at 12 months and baseline. $_c\beta$: crude standardized coefficients; $_a\beta$: adjusted standardized coefficients; SE: standard error. P-values were from linear regression analysis.

Supplemental materials Table S3. Influence of various anthropometric and biological variables on total hip aBMD variation at 12 months.

			% relative variation (Δ 12m-baseline/baseline) of the total hip aBMD					
**Continuous variables expressed as % relative variation (Δ 12m-baseline/baseline)**		**N**	**Univariate analysis**			**Multivariate analysis (n=71)**		
			$_c\beta$	SE	**P-value**	$_a\beta$	SE	**P-value**
**Age at baseline**		74	0.030	0.041	0.461			
**Weight**		74	0.237	0.052	**<0.001**	0.178	0.056	**0.002**
**BMI**		74	0.236	0.052	**<0.001**			
**Calcium**		74	0.160	0.162	0.329			
**Phosphorus**		74	-0.007	0.026	0.790	0.038	0.024	0.119
**iPTH**		74	0.045	0.015	**0.004**	0.043	0.015	**0.005**
**25(OH)D3**		74	-0.003	0.012	0.793			
**Albumin**		72	0.040	0.069	0.570			
**Osteocalcin**		73	-0.01	0.008	0.231	-0.017	0.008	**0.025**
**CTX**		73	0.001	0.002	0.560			
**Periostin**		74	0.003	0.024	0.914			
**Sclerostin**		74	0.008	0.026	0.766			
**Semaphorin**		74	0.052	0.03	0.084	0.036	0.026	0.175
**Categorical variables at baseline**								
**Gender**	**Male**	18/74	1.885	1.124	0.098	1.578	1.077	0.148
	**Female**	56/74						
**Hypertension**	**Yes**	31/74	-0.083	0.996	0.934			
	**No**	43/74						
**Type 2 diabetes mellitus**	**Yes**	14/74	1.063	1.248	0.397			
	**No**	60/74						
**Sleep apnoea**	**No**	27/74	0.908	1.015	0.374	1.673	0.941	0.080
	**Yes**	47/74						

Legend: BMI: body mass index, iPTH: intact parathyroid hormone; 25(OH)D3: 25 vitamin D; CTX: type I-C telopeptide breakdown products. Δ 12m-baseline/baseline represents the % relative difference between values at 12 months and baseline. $_c\beta$: crude standardized coefficients; $_a\beta$: adjusted standardized coefficients; SE: standard error. P-values were from linear regression analysis.

Supplemental materials Table S4. Influence of various anthropometric and biological variables on total hip aBMD variation at 24 months.

		**% relative variation (Δ 24m-baseline/baseline) of the total hip aBMD**					
**Continuous variables expressed as % relative variation (Δ 12m-baseline/baseline)**	**N**	**Univariate analysis**			**Multivariate analysis (n=53)**		
		**$_c\beta$**	SE	**p-value**	**$_a\beta$**	SE	**p-value**
**Age at baseline**	60	0.02	0.051	0.703			
**Weight**	60	0.209	0.056	**<0.001**	0.170	0.054	**0.003**
**BMI**	58	0.206	0.057	**0.001**			
**Calcium**	60	-0.101	0.188	0.592			
**Phosphorus**	60	-0.014	0.032	0.675			
**iPTH**	60	-0.001	0.012	0.917			
**25(OH)D3**	60	0.009	0.01	0.385			
**Albumin**	58	-0.021	0.07	0.761			
**Osteocalcin**	58	-0.039	0.009	**<0.001**	-0.030	0.009	**0.001**
**CTX**	59	-0.004	0.003	0.227			
**Periostin**	59	-0.014	0.027	0.595			
**Sclerostin**	59	0.067	0.035	0.06	0.050	0.029	0.087
**Semaphorin**	55	0.051	0.021	**0.021**	0.043	0.017	**0.014**
**Categorical variables at baseline**							
**Gender** — **Male** / **Female**	16/60<br>44/60	0.906	1.346	0.504			
**Hypertension** — **Yes** / **No**	27/60<br>33/60	-0.843	1.196	0.484			
**Type 2 diabetes mellitus** — **Yes** / **No**	15/60<br>45/60	-0.707	1.377	0.610			
**Sleep apnoea** — **No** / **Yes**	22/60<br>38/60	0.133	1.24	0.915			

Legend: BMI: body mass index, iPTH: intact parathyroid hormone; 25(OH)D3: 25 vitamin D; CTX: type I-C telopeptide breakdown products. Δ 12m-baseline/baseline represents the % relative difference between values at 12 months and baseline. $_c\beta$: crude standardized coefficients; $_a\beta$: adjusted standardized coefficients; SE: standard error. P-values were from linear regression analysis.

Supplemental materials Table S5. Influence of various anthropometric and biological variables on lumbar spine aBMD variation at 12 months.

		% relative variation (Δ 12m-baseline/baseline) of the lumbar spine aBMD					
**Continuous variables expressed as % relative variation (Δ 12m-baseline/baseline)**	**n**	**Univariate analysis**			**Multivariate analysis (n=68)**		
		**$_c\beta$**	SE	**p-value**	**$_a\beta$**	SE	**p-value**
**Age at baseline**	72	-0.003	0.041	0.95			
**Weight**	72	0.193	0.054	**0.001**	0.173	0.048	**<0.001**
**BMI**	72	0.193	0.054	**0.001**			
**Calcium**	72	0.238	0.161	0.144	0.365	0.140	**0.012**
**Phosphorus**	72	-0.031	0.028	0.275	0.036	0.024	0.130
**iPTH**	72	-0.026	0.016	0.102			
**25(OH)D3**	72	0.002	0.012	0.846	0.016	0.001	0.099
**Albumin**	70	-0.159	0.067	**0.021**	-0.200	0.055	**0.0006**
**Osteocalcin**	71	-0.029	0.007	**<0.001**	-0.023	0.007	**0.0011**
**CTX**	71	-0.003	0.002	0.169	0.005	0.002	**0.0182**
**Periostin**	72	-0.014	0.024	0.542			
**Sclerostin**	72	0.03	0.025	0.241			
**Semaphorin**	72	-0.005	0.03	0.874			
**Categorical variables at baseline**							
**Gender** **Male**<br>**Female**	16/72<br>56/72	2.348	1.151	**0.045**			
**Hypertension** **Yes**<br>**No**	29/72<br>43/72	0.192	1.004	0.849			
**Type 2 diabetes mellitus** **Yes**<br>**No**	14/72<br>58/72	1.060	1.238	0.395	1.880	1.010	0.068
**Sleep apnoea** **No**<br>**Yes**	27/72<br>45/72	-0.274	1.017	0.788			

Legend: BMI: body mass index, iPTH: intact parathyroid hormone; 25(OH)D3: 25 vitamin D; CTX: type I-C telopeptide breakdown products. Δ 12m-baseline/baseline represents the % relative difference between values at 12 months and baseline. $_c\beta$: crude standardized coefficients; $_a\beta$: adjusted standardized coefficients; SE: standard error. P-values were from linear regression analysis.

Supplemental materials Table S6. Influence of various anthropometric and biological variables on lumbar spine aBMD variation at 24 months.

		% relative variation (Δ 24m-baseline/baseline) of the lumbar spine aBMD					
**Continuous variables expressed as % relative variation (Δ 12m-baseline/baseline)**	**n**	**Univariate analysis**			**Multivariate analysis (n=56)**		
		**$_c\beta$**	SE	**p-value**	**$_a\beta$**	SE	**p-value**
**Age at baseline**	59	0.002	0.056	0.975			
**Weight**	59	0.11	0.067	0.107	0.093	0.057	0.111
**BMI**	57	0.107	0.068	0.121			
**Calcium**	59	0.328	0.201	0.108	0.342	0.172	0.053
**Phosphorus**	59	-0.009	0.036	0.801			
**iPTH**	59	-0.032	0.013	**0.017**			
**25(OH)D3**	59	0.03	0.011	**0.007**	0.019	0.009	**0.044**
**Albumin**	57	0.11	0.079	0.166			
**Osteocalcin**	57	-0.041	0.011	**<0.001**	-0.048	0.009	**<.0001**
**CTX**	58	0.002	0.004	0.562			
**Periostin**	58	0.044	0.029	0.133	0.074	0.023	**0.002**
**Sclerostin**	58	0.062	0.038	0.110			
**Semaphorin**	54	-0.007	0.024	0.784			
**Categorical variables at baseline**							
**Gender** **Male**<br>**Female**	15/59<br>44/59	2.449	1.477	0.103	2.315	1.171	0.054
**Hypertension** **Yes**<br>**No**	26/59<br>33/59	0.221	1.326	0.868			
**Type 2 diabetes mellitus** **Yes**<br>**No**	15/59<br>44/59	1.828	1.493	0.226			
**Sleep apnoea** **No**<br>**Yes**	22/59<br>37/59	-1.328	1.35	0.329			

Legend: BMI: body mass index, iPTH: intact parathyroid hormone; 25(OH)D3: 25 vitamin D; CTX: type I-C telopeptide breakdown products. Δ 12m-baseline/baseline represents the % relative difference between values at 12 months and baseline. $_c\beta$: crude standardized coefficients; $_a\beta$: adjusted standardized coefficients; SE: standard error. P-values were from linear regression analysis.

Supplemental materials Table S7. Influence of various anthropometric and biological variables on radius aBMD variation at 12 months.

		% relative variation (Δ 12m-baseline/baseline) of the radius					
**Continuous variables expressed as % relative variation** (Δ 12m-baseline/baseline)	**n**	**Univariate analysis**			**Multivariate analysis (n=74)**		
		**$_c\beta$**	SE	**p-value**	**$_a\beta$**	SE	**p-value**
Age at baseline	74	0.013	0.015	0.387			
Weight	74	0.057	0.021	**0.008**	0.057	0.021	**0.008**
BMI	74	0.057	0.021	**0.008**			
Calcium	74	0.059	0.061	0.337			
Phosphorus	74	0.013	0.01	0.183			
iPTH	74	0.006	0.006	0.329			
25(OH)D3	74	0.004	0.005	0.334			
Albumin	72	-0.004	0.026	0.888			
Osteocalcin	73	0.002	0.003	0.452			
CTX	73	-0.0002	0.001	0.835			
Periostin	74	0.002	0.009	0.855			
Sclerostin	74	-0.005	0.01	0.607			
Semaphorin	74	0.008	0.011	0.466			
**Categorical variables at baseline**							
Gender Male<br>Female	18/74<br>56/74	0.590	0.423	0.167			
Hypertension Yes<br>No	31/74<br>43/74	0.190	0.372	0.611			
Type 2 diabetes mellitus Yes<br>No	14/74<br>60/74	0.853	0.458	0.067			
Sleep apnoea No<br>Yes	27/74<br>47/74	-0.269	0.38	0.483			

Legend: BMI: body mass index, iPTH: intact parathyroid hormone; 25(OH)D3: 25 vitamin D; CTX: type I-C telopeptide breakdown products. Δ 12m-baseline/baseline represents the % relative difference between values at 12 months and baseline. $_c\beta$: crude standardized coefficients; $_a\beta$: adjusted standardized coefficients; SE: standard error. P-values were from linear regression analysis.

Supplemental materials Table S8. Influence of various anthropometric and biological variables on radius aBMD variation at 24 months.

		% relative variation (Δ 24m-baseline/baseline) of the radius					
**Continuous variables expressed as % relative variation** (Δ 12m-baseline/baseline)	**n**	**Univariate analysis**			**Multivariate analysis (n=59)**		
		$_c$**β**	SE	**p-value**	$_a$**β**	SE	**p-value**
Age at baseline	60	-0.003	0.027	0.916			
Weight	60	0.075	0.031	**0.021**	0.090	0.029	**0.003**
BMI	58	0.072	0.031	**0.025**			
Calcium	60	0.015	0.099	0.883			
Phosphorus	60	0.009	0.017	0.605			
iPTH	60	-0.018	0.006	**0.005**	-0.018	0.006	**0.006**
25(OH)D3	60	0.005	0.005	0.399			
Albumin	58	-0.052	0.038	0.172			
Osteocalcin	58	-0.009	0.006	0.101			
CTX	59	-0.002	0.002	0.284			
Periostin	59	0.003	0.014	0.807			
Sclerostin	59	0.054	0.017	**0.003**	0.033	0.017	**0.050**
Semaphorin	55	0.013	0.01	0.198			
**Categorical variables at baseline**							
Gender Male<br>Female	16/60<br>44/60	1.008	0.698	0.154			
Hypertension Yes<br>No	27/60<br>33/60	-0.504	0.628	0.425			
Type 2 diabetes mellitus Yes<br>No	15/60<br>45/60	0.055	0.725	0.940			
Sleep apnoea No<br>Yes	22/60<br>38/60	-0.337	0.65	0.607			

Legend: BMI: body mass index, iPTH: intact parathyroid hormone; 25(OH)D3: 25 vitamin D; CTX: type I-C telopeptide breakdown products. Δ 12m-baseline/baseline represents the % relative difference between values at 12 months and baseline. $_c$β: crude standardized coefficients; $_a$β: adjusted standardized coefficients; SE: standard error. P-values were from linear regression analysis
